# A randomized controlled trial of an ambulatory approach versus the hospital-based approach in managing suspected obstructive sleep apnea syndrome

**DOI:** 10.1038/srep45901

**Published:** 2017-04-04

**Authors:** David S. Hui, Susanna S. Ng, Kin-Wang To, Fanny W. Ko, Jenny Ngai, Ken K. P. Chan, Wing-Ho Yip, Tat-On Chan, Karen Yiu, Wilson W. S. Tam

**Affiliations:** 1 Dept of Medicine & Therapeutics, The Chinese University of Hong Kong, Hong Kong; 2 SH Ho Sleep Apnoea Management Center, The Chinese University of Hong Kong, Hong Kong; 3 Alice Lee Centre for Nursing Studies, National University of Singapore, Singapore.

## Abstract

Comparisons of home-based versus hospital-based approach in managing patients with suspected obstructive sleep apnoea syndrome(OSAS). A prospective, controlled CPAP parallel study of new referrals with suspected OSAS randomized into group A) home-based or B) hospital-based approach. Following detection of AHI ≥ 15/hr by Embletta sleep study (group A) or polysomnography (group B), patients received CPAP for 3 months after an overnight autoCPAP titration at home or in hospital respectively. Modified intention-to-treat analysis of those with AHI ≥ 15/hr on CPAP (n = 86 vs 86) showed no difference in Epworth sleepiness score, the primary endpoint, but greater improvement in Sleep-Apnoea-Quality-of-Life-Index[difference 0.3,(95%CI 0.02, 0.6), p = 0.033] at 3 months in group A. The mean costs for the patients in group A and group B were HK$8479(989) and HK$22,248(2407) respectively. The mean difference between groups was HK$-13,769(USD 1770 equivalent) per patient with 95% CI. (−14324, −13213), p < 0.001. The waiting time of patients with AHI ≥ 15/hr who were started on CPAP treatment from the first clinic consultation to the diagnostic sleep test, autoCPAP titration, and CPAP treatment was 189.6, 148.8 and 145.0 days shorter in group A than group B respectively. Home-based approach is non-inferior to hospital-based approach in managing patients with suspected OSAS, with shorter waiting time, and substantial cost savings.

Untreated obstructive sleep apnoea syndrome (OSAS) causes daytime sleepiness, cognitive function impairment and is associated with hypertension, atrial fibrillation, heart failure, sudden death, and stroke^1^. Attended polysomnography (PSG) is the standard investigation for patients with suspected OSAS, but the waiting time is often lengthy^2^. In recent years, several portable monitoring devices have been validated as useful alternatives of PSG, with the potential advantages of reducing the waiting time and healthcare cost^3^,^4^. The algorithms of managing patients with suspected OSAS were discussed by the American Academy of Sleep Medicine (AASM), and other professional societies^5^,^6^. The AASM task force has recommended that at a minimum, portable monitoring device must record airflow, respiratory effort, and blood oxygenation^7^.

Several research groups have examined different models of care involving initial home-based sleep test in diagnosing OSAS, identifying patients who may benefit from continuous positive airway pressure (CPAP), and reducing the need for hospital-based PSG and CPAP titration with encouraging results^8^,^9^,^10^,^11^,^12^,^13^,^14^. However very few studies have addressed healthcare costs, with conflicting results^13^,^14^, and there are unresolved issues as to what disease management models based on economic outcome and what patient population would be appropriate for ambulatory management^15^,^16^.

This comprehensive study aimed to assess the role of an ambulatory approach with home diagnostic sleep test followed by home autoCPAP titration versus the hospital-based approach in managing patients with suspected OSAS with reference to 1) Improvement of subjective sleepiness; 2) Quality of life; 3) Cognitive function; 4)CPAP usage; and 5) Healthcare costs.

We hypothesized that the ambulatory approach would be non-inferior to the conventional approach in managing patients with OSAS in terms of clinical outcome but the former approach would lead to substantial cost savings.

## Methods

### Subjects and Methods

We conducted a prospective, randomized, controlled CPAP parallel study on new referrals to the Respiratory Clinic, Prince of Wales Hospital, Shatin, with suspected OSAS from 25 September 2013 to 31 August 2014. OSAS was defined by apnea-hypopnea index (AHI) ≥ 5/hr of sleep plus excessive daytime sleepiness or two of the following symptoms: choking or gasping during sleep, recurrent awakenings from sleep, unrefreshed sleep, daytime fatigue, and impaired concentration^17^.

### Inclusion criteria

All patients, age 18–80 years, with suspected OSAS underwent assessment at the clinic with the Epworth sleepiness score (ESS)^18^ and symptoms evaluation. Patients who had ESS score >9 or at least two OSAS symptoms as described above were invited to join the study.

*Exclusion criteria* included patients with (a) unstable cardiovascular diseases (e.g. recent unstable angina, myocardial infarction, stroke within the previous 6 months or severe left ventricular failure), (b) neuromuscular disease affecting respiratory muscles, (c) moderate to severe respiratory disease or documented hypoxemia or awake SaO2 <92% or (d) psychiatric disease that limited the ability to give informed consent.

They were randomized by a random table into either group (A) home-based management approach or group (B) hospital-based management approach by a third party not involved in the trial. In both groups, the patients first went through the evaluation phase and if they had symptoms of OSAS with AHI ≥ 15/hr on home Embletta sleep test (Group A) or hospital-based PSG (Group B), they would then be enrolled to the CPAP outcome study (i.e. the second phase).

#### Group A

Patients in group A underwent a level 3 home sleep study with the Embletta device (Medcare, Iceland), which had been validated against hospital-based PSG in the Chinese population in Hong Kong^4^. The Embletta^TM^ PDS is a pocket-sized, digital, multi-channel recording device that measures airflow through a nasal cannula connected to a pressure transducer, providing an AHI based on recording time. It detects respiratory and abdominal efforts through the effort sensor and can differentiate between obstructive and central events. Built-in position sensors can differentiate supine from non-supine events. Patients were instructed by nurses how to operate the Embletta device for the sleep recording and estimate their time of sleep^4^.

Respiratory events are scored when desaturations of ≥4% occurs in the absence of moving artefacts and irrespective of co-existing changes in snoring or heart rate. The Embletta^TM^ PDS default settings for apneas and hypopneas were used in this study. An apnea was defined as a decrease in airflow by 80% of baseline for ≥10 seconds. A hypopnea was defined as a decrease in airflow by 50% of baseline for ≥10 seconds. The Embletta^TM^ PDS AHI used for analysis was automatically analyzed by the Embletta^TM^ PDS software^4^.

Following detection of OSAS with AHI ≥ 15/hr on home Embletta sleep study, each patient was interviewed by the physician on duty and invited to participate in the overnight home autoCPAP titration study. We offered symptomatic patients with AHI ≥ 15/hr early CPAP treatment as this group of patients were most likely to be adherent to CPAP^19^, and had the greatest risk of adverse health outcomes related to OSAS^20^,^21^. Those who had failed the Embletta sleep study were arranged to have a second home Embletta sleep study. Patients who were symptomatic of OSAS with a negative home Embletta sleep study were arranged to have a hospital-based PSG. Patients with AHI ≥ 15/hr were offered a basic CPAP education package by nurses and a 30-minute CPAP acclimatization trial with the AutoSet device (ResMed, NSW, Australia) in the afternoon at the clinic before an unattended overnight autoCPAP titration study at home (22).

#### Group B

Overnight PSG(Alice LE, Respironics, USA) was performed as at the hospital for every subject in group B recording electroencephalogram, electro-oculogram, submental electromyogram, bilateral anterior tibial electromyogram, electrocardiogram, chest and abdominal wall movement by inductance plethysmography, airflow measured by a nasal pressure transducer [PTAF2, Pro-Tech, Woodinville, WA, USA] and supplemented by oronasal airflow thermistor, plus finger pulse oximetry. (22) Apnea was defined as cessation of airflow for >10 seconds with drop in the peak thermal sensor excursion by ≥90% of baseline whereas hypopnea as a reduction of nasal pressure airflow of ≥30% of baseline for >10 seconds plus an oxygen desaturation of ≥4% (23).

Patients with AHI ≥ 15/hr on PSG underwent attended autoCPAP titration in the hospital setting after receiving a basic CPAP education package by nurses and a 30-minute CPAP acclimatization trial with the AutoSet device in the afternoon at the clinic (22).

The CPAP level for both groups A and B was set at the median 95^th^ centile pressure needed during autoCPAP titration at home (group A) and hospital setting (group B) to abolish snoring, obstructive respiratory events and airflow limitation. The patients in both groups were followed up at the CPAP clinic at 1 month and at 3 months by nurses to manage any problem with the CPAP device or mask fit and monitor the CPAP compliance. Subjects in both groups were prescribed nasal CPAP units with time clocks to assess objective CPAP usage (run time). In both arms of care, extra nursing consultations and phone advice were possible and were recorded.

Those with mild OSAS (AHI 5–15/hr) in both group A and group B were advised by physicians on treatment alternatives such as dental device, lifestyle modifications^22^ if appropriate or a trial of CPAP if agreeable but they were not included in the CPAP outcome study.

### Outcome assessment

Prior to commencement of home or conventional CPAP treatment, all patients underwent baseline assessment including ESS^18^, sleep-apnoea-specific-quality-of-life index(SAQLI)^23^, and cognitive function tests^24^, and these were reassessed at 3 months after CPAP treatment.

The ESS is a questionnaire specific to symptoms of daytime sleepiness and the patients are asked to score the likelihood of falling asleep in eight different situations with different levels of stimulation, adding up to a total score of 0 to 24 (18).

The SAQLI has 35 questions organized into four domains: daily functioning, social interactions, emotional functioning and symptoms with a fifth domain, treatment-related symptoms, to record the possible negative impacts of treatment (25).

Cognitive function tests including trail-making, digit-symbol, digit-span and Stroop colour testing were performed to provide objective evidence for improvement in daytime function on CPAP treatment, as in our previous study. (22) The trail-making test estimates the minimum time required to connect a structured number sequence and the lower the score, the better the performance. The digit symbol and span tests involve the immediate memory and recall of number sequences while the stroop colour test evaluates the correct matching of colour and their corresponding characters. For the Stroop color, digit-symbol, and digit-span tests, a higher score indicated superior performance (22).

The primary endpoints included the difference in changes in ESS, SAQLI, cognitive function tests, objective CPAP usage after 3 months of CPAP treatment between the 2 groups. Secondary endpoints included difference in healthcare costs.

### Sample size estimation

The study was powered to demonstrate non-inferiority of the ambulatory approach versus the conventional approach with respect to change in ESS, the primary outcome measure. A sample size of 86 patients in each group achieved 90% power to detect non-inferiority using a one-sided, independent samples t-test. The margin of equivalence was set at 2, i.e. a difference of this size or less on the ESS scale was not considered to be clinically important. The one-sided significance level (alpha) of the test was set at 0.025. It was assumed that the common standard deviation for change in ESS in moderate-severe OSAS was 4^25^. As our respiratory clinic generally attracted 75% of patients with at least moderate OSAS, in order to allow for 10% drop-out over the course of the study, we proceeded to recruit at least 130 patients in each group.

### Statistical analysis

Data were analysed on an intention-to-treat (ITT) basis comparing the two groups in terms of demographics, and waiting time for diagnostic tests, CPAP titration and treatment by including all patients randomized. Data were analysed on a modified ITT basis by including patients with AHI ≥ 15/hr starting CPAP therapy in both groups. For comparisons of baseline characteristics, health economics, CPAP compliance and magnitude on change in outcomes (treatment minus baseline) between the 2 groups, independent samples t-test was used for continuous variables and chi-squared test for categorical variables. To compare the measurements before and after CPAP treatment, paired samples t-test was used. Data analysis was performed with IBM SPSS (IBM SPSS Statistics for Windows, Version 22.0. Armonk, NY: IBM Corp). As a sensitivity analysis, treatment per-protocol (TPP) analysis was conducted for those with AHI ≥ 15/hr and had completed the 3-month CPAP outcome study.

### Cost-effectiveness analysis

Costs were divided into 2 parts, namely within-study costs and implementation costs in practice^11^,^15^. Cost consequences analysis, i.e. a comparison of costs with different outcomes, was conducted^26^. Within study costs included the resource use and cost over the study period from randomization (eg, staff cost, CPAP device and its education package, attended training for the patients at clinic or hospital, overnight testing with PSG versus home monitoring, any Embletta recording failure and need for repeating the test, productivity loss due to sick leave of patients, and etc). The implementation cost in practice not only included the within-study cost but also other factors comparable to the reality (eg, the prevalence of patients with different severity, the waiting time for having PSG or unattended home CPAP titration, etc) (15). All costs associated with the continuum of care (CPAP equipment and disposables, dental appliance or surgical intervention, physician visits, etc) were taken into account (15). More details of the cost-effectiveness analyses are available in the Supplementary Information.

This study protocol was approved by the Ethics Committees of the Chinese University of Hong Kong (CREC-2011.215-T) and registered at ClinicalTrials.gov(Identifier: NCT01828216) on 8 April 2013. The methods were carried out in accordance with the relevant guidelines and regulations. Written informed consent was obtained from all subjects enrolled in this study.

## Results [mean(SD) unless stated otherwise]

### Baseline Characteristics

**Figure 1:**
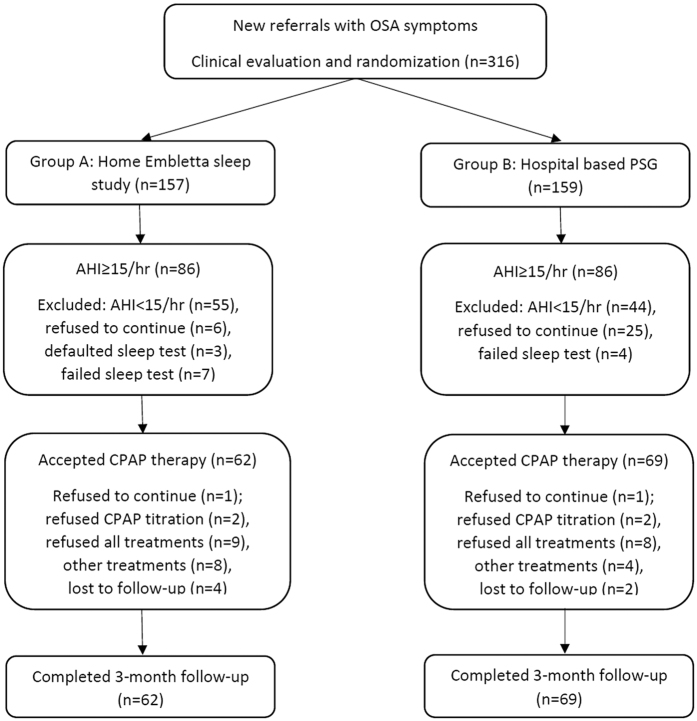
Consort diagram of participants randomized to the home-based versus hospital-based management pathways. Data were analysed on an intention-to-treat (ITT) basis comparing the two groups in terms of demographics, and waiting time for diagnostic tests, CPAP titration and treatment by including all patients randomized. Data were analysed on a modified ITT basis by including patients with AHI ≥ 15/hr starting fixed CPAP therapy in both groups whereas treatment per-protocol (TPP) analysis was conducted for those with AHI ≥ 15/hr who had completed the 3-month CPAP outcome study. * In group A with AHI ≥ 15/hr, 17 out of 86 patients failed the first home sleep test and had to undergo the second home sleep test which was successful. In group B with AHI ≥ 15/hr, 2 out of 86 patients had to undergo PSG twice. Seven out of 157 and 4 out of 159 in group A and group B “failed” sleep tests twice respectively due to either technical problems or poor sleep (total sleep time <0034 hrs) and the 7 patients in group A proceeded to PSG. # In group B, 25 patients refused to continue the study after randomization as the waiting time for polysomnography was too long.

In the first phase, altogether 316 subjects were recruited and randomized into group A (n = 157) and group B (n = 159) as shown in Fig. 1. Eighty six subjects were found to have AHI ≥ 15/hr in both Group A and B. Sixty two subjects in Group A and 69 subjects in Group B completed the 3-month follow-up. Table 1 showed the demographic data between Group A and B. The two groups were similar for most of the variables including the oxygen desaturation index (ODI) except for lower AHI and ESS in Group A (Table 1).

The waiting time of patients with AHI ≥ 15/hr who were started on CPAP treatment from the first clinic consultation to the diagnostic sleep test, autoCPAP titration, and CPAP treatment was 59.3 (35.2), 117.3 (41.0) and 154.7 (69.5) days for group A versus 248.9 (186.0), 266.1 (192.5) and 299.7 (198.7) days for group B respectively. All the waiting time for sleep tests and treatment in group A was significantly shorter than group B (p < 0.001) (Table 2).

Using a modified ITT analysis of those with AHI ≥ 15/hr (n = 86 vs 86), group A had greater improvement in SAQLI at 3 months [difference 0.3, (95% CI 0.02, 0.6), p = 0.033]. There were no significant differences in ESS and most of the cognitive function tests except for greater improvement in stroop colour testing with words in group A [difference 2.6, (95% CI 0.1, 5.1), p = 0.038] (Table 3). The results using TPP approach were similar to those analyzed by the modified ITT approach (Tables 4 and 5).

Since home sleep test could underestimate the AHI, additional analysis was performed by comparing moderate OSA (AHI 15–30/hr by home Embletta) in group A with severe OSA (AHI > 30/hr by PSG) in group B by modified ITT (n = 43 vs n = 48) and TPP (n = 33 vs n = 39) respectively. Apart from some differences in trail making with words and stroop colour, differences in other endpoints were not significant (supplemental file Table S2a–d).

#### Healthcare costs

For those who had dropped out after commencement of CPAP, a last observation carried forward approach was used to impute their wage loss and transportation expense. The approach should be acceptable in this scenario as it is expected their salary and the route to go to hospital would be the same. Following modified ITT analysis, the mean (SD) costs for the patients in group A (n = 86) and group B (n = 86) were HK$8479 (989) and HK$22,248 (2407) respectively. The mean difference between groups was HK$-13,769 per patient with 95% CI. (−14324, −13213), p < 0.001.

Status of those with AHI < 15/hr (n = 98) who were excluded from the 3-month CPAP outcome study is shown in the Supplementary Table S1.

Patients who were symptomatic of OSAS with a negative home Embletta sleep study (n = 10) were arranged to have a hospital-based PSG, which revealed that 2 patients had AHI ≥ 15/hr. Those who had failed the first Embletta sleep study (n = 7) were arranged to have a second home Embletta sleep study. A “failed” home study means that a case could not complete the home sleep test successfully due to either technical problems (poor signals) or poor sleep (TST < 4 hrs), despite having repeated the test. Of these 7 patients who had undergone PSG, 3 were found to have AHI ≥ 15/hr. Thus altogether 10 + 7 = 17 patients in group A finally required PSG, with a waiting time of 384.4 (312.4) days for PSG.

## Discussion

This randomized controlled trial (RCT) compared a home-based approach using a validated level 3 sleep diagnostic device^4^ for diagnosis followed by one night of autoCPAP titration against the hospital-based sleep laboratory approach in managing clinic patients with suspected OSAS. The waiting time of patients with AHI ≥ 15/hr who were started on CPAP treatment from the first clinic consultation to the diagnostic sleep test, autoCPAP titration, and CPAP treatment was 189.6, 148.8 and 145.0 days shorter in group A than in group B respectively. The much longer waiting time for PSG and autoCPAP titration in the hospital setting was due to the limited number of hospital beds designated for sleep medicine service whereas the home-based approach offered much more flexibility.

Using a modified ITT analysis of those with AHI ≥ 15/hr who had commenced home CPAP treatment, Group A had slightly greater improvement in SAQLI and stroop colour testing with words at 3 months whereas there was no difference in other cognitive function tests between the 2 groups. As importantly, there was significant cost saving of HK$13,769 (USD1770 equivalent) per patient in favour of group A. However, whether there is significant cost saving using home management approach would depend on the local healthcare system and costs. A RCT in Spain has shown that patients with a high probability of OSAS could be diagnosed and treated in a home setting, with a high level of CPAP compliance and lower cost (€590 vs €894 vs €644) than using either a hospital-based approach or home respiratory polygraphy/hospital follow-up.(13) In contrast, another RCT in the USA has shown cost saving of US$264(95% CI $39, $496, P = 0.02) in favor of home-based management from the patient’s perspective but US$40 (95% CI -$213, $142, P = 0.66) in favor of the laboratory-based arm under the base case from the provider perspective^14^.

Our study findings add more strength to the growing literature that the ambulatory approach is an alternative strategy in managing clinic patients with suspected OSAS. In a RCT conducted by Skomro *et al*.^8^ comparing an ambulatory approach (home-based level 3 testing followed by one week of autoCPAP titration and then fixed-pressure CPAP based on 95^th^ centile pressure) vs in-laboratory PSG and CPAP titration, the laboratory approach did not lead to superior four-week outcomes in sleepiness scores, sleep quality, quality of life, blood pressure, and CPAP adherence. Another RCT by Berry *et al*.^9^, comparing a level 3 portable monitoring(Watch PAT-100) and autoCPAP titration(2–3 nights) vs PSG for the diagnosis and treatment of OSAS, has shown that the former approach resulted in CPAP adherence and clinical outcomes similar to the one using PSG. In a RCT that compared standard PSG against ambulatory CPAP titration (autoCPAP titration for 1 week followed by CPAP set at 95^th^ centile pressure) in high-risk patients identified by a diagnostic algorithm involving symptom score and a level 3 portable sleep diagnostic device, Mulgrew *et al*.^10^ have shown no advantage of PSG over the ambulatory approach in terms of diagnosis, CPAP titration(AHI on CPAP), ESS, and SAQLI over 3 months whereas adherence to CPAP therapy was better in the ambulatory group. A nurse-led model of care by Antic *et al*.^11^ using 4 nights of home auto-adjusting device after overnight home oximetry to set therapeutic CPAP has demonstrated non-inferior results(change in ESS, CPAP adherence at 3 months) to physician-directed care, which involved two laboratory PSG to diagnose and treat patients with moderate to severe OSAS. Kuna *et al*.^12^, who randomized patients with suspected OSAS to either home testing with the Embletta device followed by at least 3 nights of autoCPAP titration or laboratory-based management, found that functional outcome and treatment adherence in patients evaluated through the home testing approach was not clinically inferior to that in patients receiving standard in-laboratory PSG.

Pressure for alternative approaches to the in-laboratory/hospital management of patients with OSAS will continue to increase given the cost of PSG, the limited number of hospital or laboratory-based facilities and the growing demand for more rapid access to testing^16^. Since 2007, the AASM has approved the use of home portable monitoring and recommended that unobserved registers with type 2–3 monitors be used as an alternative to PSG diagnosis^7^, and unattended autoCPAP titration to determine a fixed CPAP treatment pressure in patients with a high probability of moderate-to-severe OSAS without significant medical comorbidities^27^.

It is important to shorten the waiting time for sleep investigation and treatment especially for those with a high clinical probability of OSAS, as many studies have shown that untreated OSA is associated with increased risks of hypertension^28^, platelet activation^29^, diastolic dysfunction^30^, mortality^20^,^31^,^32^,^33^, sudden death^21^,^34^, and stroke^21^,^35^,^36^. CPAP has been shown to reduce systemic blood pressure^37^,^38^, daytime sleepiness^39^, risk of driving accidents^40^, and platelet activation^29^ although it remains to be seen by proper RCTs whether it may reduce mortality in patients with OSAS^41^.

One limitation of this study was that the Embletta diagnostic device had underestimated the AHI because it only recorded the number of obstructive events/hour of recording instead of actual sleep time although the ODI in both groups was comparable. Moreover, only 62 and 69 out of 86 patients completed 3 months of CPAP treatment in group A and group B respectively. Without adequate contact with health professionals, more patients in group A were likely not inclined to follow-up care.

In conclusion, the ambulatory approach for diagnosis and treatment was non-inferior to the conventional hospital-based approach in managing clinic patients in Hong Kong with suspected OSAS in terms of CPAP usage and improvement of some clinical endpoints, with the advantages of much shorter waiting time, and substantial cost savings.

## Additional Information

**How to cite this article**: Hui, D. S. *et al*. A randomized controlled trial of an ambulatory approach versus the hospital-based approach in managing suspected obstructive sleep apnea syndrome. *Sci. Rep.* **7**, 45901; doi: 10.1038/srep45901 (2017).

**Publisher's note:** Springer Nature remains neutral with regard to jurisdictional claims in published maps and institutional affiliations.

## Supplementary Material

Supplementary File

## Figures and Tables

**Table 1:** Baseline characteristics and time to investigation and treatment.

	Home (n = 157)	Hospital (n = 159)	P-value
Age	51.0 (12.9)	52.1 (11.3)	0.403
Male sex [n(%)]	107 (68.2%)	118 (74.2%)	0.264
Current smoker [n(%)]	20 (12.8%)	24 (15.1%)	0.691
Current drinker [n(%)]	26 (23.0%)	31 (26.3%)	0.765
Congestive Heart Failure [n(%)]	6 (3.8%)	5 (3.1%)	0.769
Diabetes Mellitus [n(%)]	25 (15.9%)	30 (18.9%)	0.554
Hypertension [n(%)]	72 (45.9%)	86 (54.1%)	0.177
Stroke [n(%)]	5 (3.2%)	2 (1.3%)	0.281
Ischemic Heart Disease [n(%)]	9 (5.7%)	11 (6.9%)	0.818
Hyperlipidemia [n(%)]	32 (20.4%)	32 (20.1%)	1.000
BMI (kg/m^2^)	27.4 (5.3)	28.3 (4.6)	0.115
Neck circumference (cm)	38.6 (3.6)	39.6 (3.8)	**0.013**
Waist circumference (cm)	95.4 (12.1)	97.3 (11.2)	0.171
Hip circumference (cm)	102.2 (9.5)	101.6 (8.1)	0.527
ESS (0–24)	11.0 (5.6)	9.6 (5.4)	**0.025**
AHI (events/hr)	24.1 (20.7)	30.8 (27.5)	**0.023**
ODI (events/hr)*	21.7 (19.8)	22.6 (23.4)	0.724

N = 157 (group A, home-based) vs 159 (group B, hospital-based).

*Oxygen desaturation index (ODI) refers to the number of times per hour of sleep that the blood oxygen level dropped by at least 4% from baseline.

**Table 2:** Baseline characteristics and time to investigation and treatment (n = 86 vs 86, modified ITT analysis).

	Home (n = 86)	Hospital (n = 86)	P-value
Age	53.7 (12.2)	52.1 (11.4)	0.374
Male sex [n(%)]	61 (70.9%)	73 (84.9%)	**0.042**
Current smoker [n(%)]	10 (11.6%)	15 (17.4%)	0.545
Current drinker [n(%)]	14 (21.5%)	20 (30.8%)	0.407
Congestive Heart Failure [n(%)]	4 (4.7%)	4 (4.7%)	1
Diabetes Mellitus [n(%)]	17 (19.8%)	19 (22.1%)	0.852
Hypertension [n(%)]	49 (57.0%)	48 (55.8%)	1
Stroke [n(%)]	4 (4.7%)	2 (2.3%)	0.682
Ischemic Heart Disease [n(%)]	7 (8.1%)	7 (8.1%)	1
Hyperlipidemia [n(%)]	21 (24.4%)	14 (16.3%)	0.256
BMI (kg/m^2^)	28.6 (5.6)	28.6 (4.4)	0.982
Neck circumference (cm)	39.3 (3.6)	40.5 (3.9)	**0.047**
Waist circumference (cm)	99.0 (12.5)	98.9 (10.3)	0.939
Hip circumference (cm)	104.4 (10.2)	102.1 (7.7)	0.094
ESS (0–24)	11.9 (5.7)	9.9 (5.5)	**0.017**
AHI (events/hr)	36.4 (19.2)	42.7 (26.2)	0.077
Time from 1^st^ consultation to diagnostic sleep test (days)	59.3 (35.2)	248.9 (186.0)	<**0.001**
ODI (events/hr)*	33.0 (19.1)	31.6 (24.1)	0.680
Time from 1^st^ consultation to CPAP titration (days)	117.3 (41.0)	266.1 (192.5)	<**0.001**
Time from 1^st^ consultation to CPAP treatment (days)	154.7 (69.5)	299.7 (198.7)	<**0.001**

*Oxygen desaturation index (ODI) refers to the number of times per hour of sleep that the blood oxygen level dropped by at least 4% from baseline.

**Table 3:** Outcomes (modified ITT analysis, n = 86 vs 86).

	Home (n = 86)	Hospital (n = 86)	Difference (95% CI)	P-value (between group)
ESS				
Baseline	11.9 (5.7)	9.9 (5.5)	2.1 (0.4, 3.7)	**0.017**
3 months	8.5 (5.5)	7.7 (5.0)	0.8 (−0.8, 2.4)	0.331
Difference	−3.5 (5.0)	−2.2 (5.2)	−1.3 (−2.8, 0.3)	0.105
P-value (within group)	<**0.001**	<**0.001**		
SAQLI				
Baseline	4.5 (1.0)	4.7 (1.0)	−0.2 (−0.5, 0.1)	0.190
3 months	4.7 (1.0)	4.6 (1.0)	0.1 (−0.2, 0.4)	0.582
Difference	0.2 (1.0)	−0.1 (0.8)	0.3 (0.02, 0.6)	**0.033**
P-value (within group)	0.070	0.260		
Digital span score				
Baseline	18.0 (4.4)	18.2 (4.4)	−0.2 (−1.5, 1.2)	0.792
3 months	17.8 (3.9)	18.3 (4.4)	−0.4 (−1.7, 0.8)	0.494
Difference	−0.2 (2.0)	0.1 (2.7)	−0.3 (−1.0, 0.5)	0.484
P-value (within group)	0.460	0.749		
Digital symbol score				
Baseline	44.8 (16.7)	49.5 (16.2)	−4.7 (−9.7, 0.3)	0.792
3 months	46.8 (17.1)	52.9 (17.5)	−6.1 (−11.4, −0.9)	**0.022**
Difference	2.0 (5.3)	3.5 (5.8)	−1.5 (−3.2, 0.2)	0.089
P-value (within group)	**0.001**	<**0.001**		
Trail making				
Baseline	47.6 (32.4)	39.0 (19.8)	8.6 (0.5, 16.8)	**0.038**
3 months	44.5 (32.7)	35.2 (18.1)	9.3 (1.3, 17.4)	**0.023**
Difference	−3.1 (8.8)	−3.8 (9.3)	0.7 (−2.1, 3.4)	0.625
P-value (within group)	**0.002**	<**0.001**		
Trail making (with words)				
Baseline	70.7 (47.9)	57.5 (37.2)	13.2 (0.1, 26.2)	**0.048**
3 months	66.4 (47.1)	55.0 (35.9)	11.3 (−1.4, 24.1)	0.081
Difference	−4.4 (10.8)	−2.5 (17.4)	−1.9 (−6.2, 2.5)	0.406
P-value (within group)	<**0.001**	0.189		
Stroop colour testing				
Baseline	70.8 (19.5)	75.1 (17.0)	−4.3 (−9.7, 1.3)	0.129
3 months	72.3 (20.2)	76.0 (17.1)	−3.7 (−9.4, 2.0)	0.202
Difference	1.6 (6.7)	1.0 (7.6)	0.6 (−1.6, 2.8)	0.591
P-value (within group)	**0.034**	0.244		
Stroop colour testing (with words)				
Baseline	38.5 (15.1)	42.5 (14.2)	−4.0 (−8.5, 0.5)	0.077
3 months	42.2 (16.5)	43.6 (13.5)	−1.4 (−6.0, 3.2)	0.550
Difference	3.8 (8.3)	1.1 (7.9)	2.6 (0.1, 5.1)	**0.038**
P-value (within group)	<**0.001**	0.191		

**Table 4:** CPAP outcomes & time to investigation and treatment (CPAP users only, n = 62 vs 69, TPP analysis).

	Home (n = 62)	Hospital (n = 69)	p-value
CPAP acceptance (%) [n(%)]	72.1%	80.2%	0.213
CPAP titration pressure (cmH_2_O)	13.3 (2.2)	12.8 (2.1)	0.252
CPAP usage at 3 months	5.0 (2.0)	3.9 (2.1)	**0.005**
% of patients who used CPAP >=4 hrs per night [n(%)]	74.2%	56.5%	**0.033**
% of patients who used CPAP >=70% of nights, >=4 hrs per night [n(%)]	74.2%	56.5%	0.123
Time from 1^st^ consultation to diagnostic sleep test (days)	54.2 (31.3)	258.9 (186.9)	<**0.001**
Time from 1^st^ consultation to CPAP titration (days)	109.0 (36.8)	274.0 (193.6)	<**0.001**
Time from 1^st^ consultation to CPAP treatment (days)	154.7 (69.5)	299.7 (198.7)	<**0.001**

**Table 5:** Other outcomes (CPAP users only, n = 62 vs 69, TPP analysis).

	Home (n = 62)	Hospital (n = 69)	Difference (95% CI)	P-value (between group)
ESS				
Baseline	12.4 (5.5)	10.2 (5.6)	2.2 (0.3, 4.1)	**0.025**
3 months	7.6 (4.8)	7.4 (5.1)	0.1 (−1.6, 1.8)	0.895
Difference	−4.8 (5.4)	−2.7 (5.7)	−2.1 (−4.0, −0.2)	**0.035**
P-value (within group)	<**0.001**	<**0.001**		
SAQLI				
Baseline	4.4 (1.0)	4.7 (0.9)	−0.3 (−0.6, 0.03)	0.078
3 months	4.7 (1.0)	4.6 (1.0)	0.1 (−0.3, 0.4)	0.621
Difference	0.3 (1.1)	−0.1 (0.9)	0.4 (0.04, 0.7)	**0.030**
P-value (within group)	0.070	0.260		
Digital span score				
Baseline	18.2 (4.3)	18.5 (4.5)	−0.3 (−1.8, 1.2)	0.701
3 months	18.0 (3.6)	18.6 (4.5)	−0.6 (−2.0, 0.8)	0.369
Difference	−0.2 (2.4)	0.12 (3.0)	−0.3 (−1.3, 0.6)	0.477
P-value (within group)	0.461	0.750		
Digital symbol score				
Baseline	46.7 (15.0)	49.8 (15.8)	−3.1 (−8.4, 2.3)	0.259
3 months	49.4 (15.2)	54.0 (17.2)	−4.6 (−10.2, 1.1)	0.111
Difference	2.7 (6.0)	4.2 (6.1)	−1.5 (−3.6, 0.6)	0.160
P-value (within group)	**0.001**	<**0.001**		
Trail making				
Baseline	42.8 (18.9)	36.8 (17.8)	6.0 (−0.3, 12.3)	0.064
3 months	38.5 (18.6)	32.2 (14.6)	6.3 (0.6, 12.1)	**0.031**
Difference	−4.3 (10.0)	−4.6 (10.1)	0.4 (−3.1, 3.8)	0.842
P-value (within group)	**0.001**	<**0.001**		
Trail making (with words)				
Baseline	63.7 (30.9)	53.5 (30.2)	10.2 (−0.4, 20.7)	0.059
3 months	57.7 (27.5)	50.5 (27.8)	7.2 (−2.3, 16.8)	0.138
Difference	−6.0 (12.3)	−3.1 (19.2)	−2.9 (−8.6, 2.7)	0.306
P-value (within group)	<**0.001**	0.189		
Stroop colour testing				
Baseline	73.2 (15.2)	76.3 (16.4)	−3.1 (−8.6, 2.4)	0.267
3 months	75.3 (16.1)	77.5 (16.5)	−2.2 (−7.8, 3.5)	0.451
Difference	2.1 (7.7)	1.2 (8.4)	0.9 (−1.9, 3.7)	0.306
P-value (within group)	**0.034**	0.244		
Stroop colour testing (with words)				
Baseline	38.0 (13.6)	43.3 (13.4)	−5.3 (−10.0, −0.6)	**0.027**
3 months	43.1 (15.6)	44.7 (12.4)	−1.6 (−6.5, 3.3)	0.529
Difference	5.1 (9.4)	1.4 (8.8)	3.7 (0.6, 6.9)	**0.020**
P-value (within group)	**<0.001**	0.192		
