# Author Correction: A randomized controlled trial of an ambulatory approach versus the hospital-based approach in managing suspected obstructive sleep apnea syndrome

**DOI:** 10.1038/s41598-019-41389-6

**Published:** 2019-04-02

**Authors:** David S. Hui, Susanna S. Ng, Kin-Wang To, Fanny W. Ko, Jenny Ngai, Ken K. P. Chan, Wing-Ho Yip, Tat-On Chan, Karen Yiu, Wilson W. S. Tam

**Affiliations:** 1 Dept of Medicine & Therapeutics, The Chinese University of Hong Kong, Hong Kong, China; 2 SH Ho Sleep Apnoea Management Center, The Chinese University of Hong Kong, Hong Kong, China; 3 Alice Lee Centre for Nursing Studies, National University of Singapore, Singapore, Singapore

Correction to: *Scientific Reports* 10.1038/srep45901, published online 04 April 2017

This Article contains errors in Table 4 where the values for ‘% of patients who used CPAP >=4 hrs per night [n(%)]’ and ‘% of patients who used CPAP >=70% of nights, >=4 hrs per night [n(%)]’ are incorrect in the ‘Home’ and ‘Hospital’ columns.

In addition, ‘Home (n = 62)’ should read ‘Home (n = 62, 72.1% of 86 patients accepted CPAP)’, and ‘Hospital (n = 69)’ should read ‘Hospital (n = 69, 80.2% of 86 patients accepted CPAP)*’.

The correct Table 4 appears below as Table 1, accompanied by a footnote which gives details of the CPAP acceptance.

**Table 1:** CPAP outcomes & time to investigation and treatment (CPAP users only, n = 62 vs 69, TPP analysis).

	Home (n=62, 72.1% of 86 patients accepted CPAP)	Hospital (n=69, 80.2% of 86 patients accepted CPAP)*	p-value
CPAP titration pressure (cmH_2_O)	13.3 (2.2)	12.8 (2.1)	0.252
CPAP usage at 3 months	5.0 (2.0)	3.9 (2.1)	**0.005**
% of patients who used CPAP >=4 hrs per night [n(%)]	46 (74.2%)	39 (56.5%)	**0.033**
% of patients who used CPAP >=70% of nights, >=4 hrs per night [n(%)]	42 (67.7%)	37 (53.6%)	0.123
Time from 1st consultation to diagnostic sleep test (days)	54.2 (31.3)	258.9 (186.9)	**<0.001**
Time from 1st consultation to CPAP titration (days)	109.0 (36.8)	274.0 (193.6)	**<0.001**
Time from 1st consultation to CPAP treatment (days)	154.7 (69.5)	299.7 (198.7)	**<0.001**

Footnote: *62 out of 86 versus 69 out of 86 patients with AHI ≥ 15/hr in the home management group and hospital-based management group respectively accepted CPAP treatment and completed 3 months of follow up. No group difference on the rate of CPAP acceptance was found, P-value = 0.213.

