# Supplementary material for: A randomized controlled trial of an ambulatory approach versus the hospital-based approach in managing suspected obstructive sleep apnea syndrome
Source: Sci Rep. 2017 Apr 4;7:45901. doi: 10.1038/srep45901 (PMC5379203; doi:10.1038/srep45901)
Supplement: Supplementary File [file srep45901-s1.doc]

"Supplement information"

**A randomized controlled trial of an ambulatory approach versus the hospital-based approach in managing suspected obstructive sleep apnea syndrome**

Hui DS, Ng SS, To KW, Ko FW, Ngai J, Chan KKP,

Yip WH, Chan TO, Yiu K, Tam W

**Cost breakdown for the ambulatory (group A) vs hospital based study (Group B) is shown as follows:**

Health Care Cost

*Hospital-based group (Group B)*:

- PSG and CPAP titration: HK$3000 per test
- Cost per patient day – General HK$4330. (1)

*Ambulatory group (Group A)*:

- Embletta sleep test and home CPAP titration: HK$500 per test
- Cost per general outpatient attendance (approximated cost for the education and borrowing the machine): HK$385. (1)

Work cost of participants and their companies (if any) for subjects in each group

- Cost of each day absent (if any) from work for the subject’s examination: Monthly salary divided by 30 days
- Cost of each day absent (if any) from work for the company: Monthly salary divided by 30 days

Transportation cost for the examination for subjects in each group

Cost on transportation for the examination of the subjects and their companies:

- Public Transportation: Fares were checked from local public transportation websites.(2-4)
- Private Driving: The route was determined retrospectively by Google Map based on the subject’ stated starting location to the hospital. The shortest distance suggested by Google Map would be used to calculate the cost including the tunnel surcharge (if any). The cost of gasoline was estimated based on the information from the Consumer Council, (5) and was set at HK$15.28 per liter. For a typical vehicle, it was assumed it could run around 15km per liter of fuel. (6) The fee of each tunnel was extracted from the Transportation Department. (7).

The total cost is the sum of the above cost for each patient. 17 patients in Group A and 2 patients in the Group B failed the first sleep test and proceeded to the second sleep test, the additional cost have been taken into consideration.

References:

1. Appendix 8 of Hospital Authority Annual Report 2013-2014, available at http://www.ha.org.hk (accessed on 30 June 2015).

2. MTR Corporation Limited. Available at <http://www.mtr.com.hk/ch/customer/tourist/index.php>

3. Kowloon Motor Bus. Available at http://www.kmb.com.hk/tc/.

4. New World First Bus and CityBus. Available at http://www.nwstbus.com.hk.

5. Auto Fuel Price Calculator, Hong Kong Consumer Council. Available at http://consumer.org.hk/fc/txtver/en_oilwatch.php.

6. The eMercedesBenz: http://emercedesbenz.com.

7. Tunnels and Bridges in Hong Kong, Transportation Department, Hong Kong SAR Government.

**Supplemental Table 1. Final status of patients with AHI <15/hr who were excluded from the CPAP outcome study**

|  | Home | Hospital | Total |
| --- | --- | --- | --- |
| Failed / Refused sleep tests | 1 | 0 | 1 |
| CPAP | 16 | 10 | 26 |
| Dental device | 10 | 8 | 18 |
| Other treatments | 5 | 2 | 7 |
| Refused all treatments | 5 | 6 | 11 |
| Unknown status/ Defaulted FU | 10 | 5 | 15 |
| No OSA | 8 | 13 | 21 |
| Total | 55 | 44 | 99 |
| P(chi-square)=0.537 |  |  |  |

**Supplemental Table S2a. Baseline characteristics and time to investigation and treatment (moderate OSA in Group A vs severe OSA in Group B, modified ITT analysis). N=43 (group A, home-based) vs 48 (group B, hospital-based)**

|  | Home (n=43) | Hospital (n=48) | P-value |
| --- | --- | --- | --- |
| Age | 53.4 (11.6) | 53.0 (11.1) | 0.860 |
|  |  |  |  |
| Male sex [n(%)] | 35 (81.4%) | 59 (91.8%) | 0.214 |
|  |  |  |  |
| Current smoker [n(%)] | 3 (7.0%) | 10 (20.4%) | 0.123 |
|  |  |  |  |
| Current drinker [n(%)] | 4 (11.8%) | 12 (33.3%) | **0.044** |
|  |  |  |  |
| Congestive Heart Failure [n(%)] | 2 (4.7%) | 2 (4.1%) | 1.000 |
|  |  |  |  |
| Diabetes Mellitus [n(%)] | 10 (23.3%) | 12 (24.5%) | 1.000 |
|  |  |  |  |
| Hypertension [n(%)] | 22 (51.2%) | 29 (59.2%) | 0.529 |
| Stroke [n(%)] | 4 (9.3%) | 2 (4.1%) | 0.413 |
| Ischemic Heart Disease [n(%)] | 2 (4.7%) | 4 (9.3%) | 0.681 |
|  |  |  |  |
| Hyperlipidemia [n(%)] | 9 (20.9%) | 8 (16.3%) | 0.601 |
|  |  |  |  |
| BMI (kg/m^2^) | 27.1 (5.5) | 29.4 (4.3) | **0.029** |
|  |  |  |  |
| Neck circumference (cm) | 39.1 (3.7) | 41.2 (2.7) | **0.002** |
|  |  |  |  |
| Waist circumference (cm) | 95.7 (12.1) | 101.2 (9.9) | **0.020** |
|  |  |  |  |
| Hip circumference (cm) | 101.7 (9.3) | 103.2 (8.0) | 0.411 |
|  |  |  |  |
| ESS (0-24) | 12.1 (5.1) | 9.4 (6.1) | **0.025** |
|  |  |  |  |
| AHI (events/hr) | 22.0 (4.7) | 59.9 (22.5) | **<0.001** |
|  |  |  |  |
| ODI (events/hr) | 18.6 (6.1) | 45.1 (23.7) | **<0.001** |
|  |  |  |  |
| Time from 1st consultation to diagnostic sleep test (days) | 57.8 (35.5) | 255.3 (196.1) | **<0.001** |
| Time from 1st consultation to CPAP titration (days) | 108.2 (41.5) | 271.8 (200.5) | **<0.001** |
| Time from 1st consultation to CPAP treatment (days) | 152.8 (63.0) | 315.2 (212.4) | **0.001** |

**Table S2b. Outcomes (modified ITT analysis, n=43 vs 49)**

|  | Home (n=43) | Hospital (n=48) | Difference (95% CI) | P-value  (between group) |
| --- | --- | --- | --- | --- |
| ESS |  |  |  |  |
| - Baseline | 12.1 (5.1) | 9.4 (6.1) | 2.7 (0.3, 5.0) | **0.025** |
| - 3 months | 8.7 (5.3) | 7.2 (5.1) | 1.5 (-0.7, 3.6) | 0.177 |
| - Difference | -3.4 (4.7) | -2.2 (5.5) | -1.2 (-3.3, 0.9) | 0.263 |
| P-value (within group) | **<0.001** | **0.008** |  |  |
|  |  |  |  |  |
| SAQLI |  |  |  |  |
| - Baseline | 4.5 (0.9) | 4.7 (1.0) | -0.2 (-0.6, 0.2) | 0.317 |
| - 3 months | 4.6 (1.0) | 4.7 (1.0) | -0.1 (-0.5, 0.3) | 0.584 |
| - Difference | 0.1 (1.0) | 0.003 (0.8) | 0.1 (-0.3, 0.5) | 0.624 |
| P-value (within group) | 0.547 | 0.977 |  |  |
|  |  |  |  |  |
| Digital span score |  |  |  |  |
| - Baseline | 18.2 (4.4) | 18.5 (4.5) | -0.3 (-2.1, 1.6) | 0.755 |
| - 3 months | 17.9 (3.8) | 18.7 (4.0) | -0.8 (-2.4, 0.9) | 0.348 |
| - Difference | -0.3 (2.3) | 0.2 (2.8) | -0.5 (-1.6, 0.6) | 0.371 |
| P-value (within group) | 0.388 | 0.651 |  |  |
|  |  |  |  |  |
| Digital symbol score |  |  |  |  |
| - Baseline | 46.3 (16.3) | 47.6 (14.9) | -1.3 (-7.9, 5.2) | 0.692 |
| - 3 months | 48.8 (17.3) | 50.8 (17.1) | -2.0 (-9.2, 5.1) | 0.572 |
| - Difference | 2.5 (5.5) | 3.2 (5.8) | -0.7 (-3.1, 1.6) | 0.534 |
| P-value (within group) | **0.005** | **<0.001** |  |  |
|  |  |  |  |  |
| Trail making |  |  |  |  |
| - Baseline | 45.0 (34.5) | 37.7 (17.9) | 7.2 (-4.0, 18.5) | 0.206 |
| - 3 months | 42.1 (34.6) | 35.1 (19.7) | 7.0 (-4.5, 18.6) | 0.231 |
| - Difference | -2.8 (7.0) | -2.6 (7.9) | -0.2 (-3.3, 2.9) | 0.893 |
| P-value (within group) | **0.011** | **0.026** |  |  |
|  |  |  |  |  |
| Trail making (with words) |  |  |  |  |
| - Baseline | 67.5 (49.8) | 55.8 (34.7) | 11.7 (-6.0, 29.5) | 0.192 |
| - 3 months | 61.6 (47.5) | 55.8 (36.9) | 5.8 (-11.8, 23.5) | 0.512 |
| - Difference | -5.9 (11.2) | 0.0 (12.9) | -5.9 (-11.0, -0.9) | **0.023** |
| P-value (within group) | **0.001** | 1.000 |  |  |
|  |  |  |  |  |
| Stroop colour testing |  |  |  |  |
| - Baseline | 72.4 (18.9) | 73.7 (13.5) | -1.3 (-8.1, 5.5) | 0.708 |
| - 3 months | 74.4 (19.9) | 74.0 (16.0) | 0.4 (-7.1, 7.9) | 0.916 |
| - Difference | 2.0 (7.2) | 0.3 (8.4) | 1.7 (-1.6, 5.0) | 0.311 |
| P-value (within group) | 0.079 | 0.811 |  |  |
|  |  |  |  |  |
| Stroop colour testing (with words) |  |  |  |  |
| - Baseline | 38.3 (16.1) | 42.1 (12.1) | -3.9 (-9.8, 2.0) | 0.194 |
| - 3 months | 42.8 (17.7) | 43.6 (11.6) | -0.8 (-7.1, 5.5) | 0.803 |
| - Difference | 4.5 (8.7) | 1.4 (6.9) | 3.1 (-0.2, 6.4) | 0.063 |
| P-value (within group) | **0.002** | 0.162 |  |  |

**Table S2c. CPAP outcomes & time to investigation and treatment (CPAP users only, n=33 vs 39, TPP analysis)**

|  | Home (n=33) | Hospital (n=39) | p-value |
| --- | --- | --- | --- |
| CPAP acceptance (%) [n(%)] | 76.7% | 81.3% | 0.616 |
|  |  |  |  |
| CPAP titration pressure (cmH2O) | 13.1 (2.5) | 12.9 (2.6) | 0.807 |
|  |  |  |  |
| CPAP usage at 3 months | 5.2 (1.8) | 3.8 (2.3) | **0.003** |
|  |  |  |  |
| % of patients who used CPAP >=4 hrs per night [n(%)] | 76.8% | 56.4% | 0.135 |
|  |  |  |  |
| % of patients who used CPAP >=70% of nights, >=4 hrs per night [n(%)] | 78.1% | 60.0% | 0.124 |
|  |  |  |  |
| Time from 1st consultation to diagnostic sleep test (days) | 54.6 (32.6) | 271.9 (199.9) | **<0.001** |
|  |  |  |  |
| Time from 1st consultation to CPAP titration (days) | 102.7 (37.1) | 284.5 (205.4) | **<0.001** |
|  |  |  |  |
| Time from 1st consultation to CPAP treatment (days) | 152.8 (63.0) | 315.2 (212.4) | **<0.001** |

**Table S2d. Outcomes (CPAP users only, n=33 vs 39, TPP analysis)**

|  | Home (n=33) | Hospital (n=39) | Difference (95% CI) | P-value  (between group) |
| --- | --- | --- | --- | --- |
| ESS |  |  |  |  |
| - Baseline | 12.1 (5.1) | 9.9 (6.2) | 2.2 (-0.5, 4.9) | 0.115 |
| - 3 months | 7.7 (4.9) | 7.2 (5.2) | 0.5 (-1.9, 2.9) | 0.680 |
| - Difference | -4.4 (5.0) | -2.7 (6.1) | -1.7 (-4.3, 0.9) | 0.207 |
| P-value (within group) | **<0.001** | **0.008** |  |  |
|  |  |  |  |  |
| SAQLI |  |  |  |  |
| - Baseline | 4.4 (1.0) | 4.6 (1.0) | -0.2 (-0.6, 0.3) | 0.490 |
| - 3 months | 4.6 (1.0) | 4.6 (0.9) | -0.04 (-0.5, 0.4) | 0.863 |
| - Difference | 0.1 (1.2) | 0.04 (0.9) | 0.1 (-0.4, 0.6) | 0.619 |
| P-value (within group) | 0.549 | 0.977 |  |  |
|  |  |  |  |  |
| Digital span score |  |  |  |  |
| - Baseline | 18.2 (4.3) | 19.0 (4.5) | -0.8 (-2.9, 1.3) | 0.458 |
| - 3 months | 17.8 (3.6) | 19.3 (3.9) | -1.4 (-3.2, 0.4) | 0.122 |
| - Difference | -0.4 (2.6) | 0.2 (3.2) | 0.7 (-2.0, 0.8) | 0.369 |
| P-value (within group) | 0.390 | 0.652 |  |  |
|  |  |  |  |  |
| Digital symbol score |  |  |  |  |
| - Baseline | 48.6 (14.3) | 49.3 (12.5) | -0.7 (-7.0, 5.6) | 0.827 |
| - 3 months | 51.8 (15.3) | 53.3 (15.0) | -1.4 (-8.6, 5.7) | 0.690 |
| - Difference | 3.2 (6.1) | 3.9 (6.3) | -0.7 (-3.6, 2.2) | 0.616 |
| P-value (within group) | **0.005** | **<0.001** |  |  |
|  |  |  |  |  |
| Trail making |  |  |  |  |
| - Baseline | 39.2 (16.2) | 33.4 (11.4) | 5.7 (-1.0, 12.5) | 0.093 |
| - 3 months | 35.5 (15.0) | 30.2 (13.5) | 5.3 (-1.4, 12.0) | 0.120 |
| - Difference | -3.7 (7.8) | -3.3 (8.7) | -0.5 (-4.4, 3.4) | 0.813 |
| P-value (within group) | **0.010** | **0.025** |  |  |
|  |  |  |  |  |
| Trail making (with words) |  |  |  |  |
| - Baseline | 60.7 (30.8) | 48.6 (18.1) | 12.1 (-0.2, 24.3) | 0.054 |
| - 3 months | 53.0 (23.0) | 48.6 (22.7) | 4.4 (-6.4, 15.1) | 0.423 |
| - Difference | -7.7 (12.2) | 0.0 (14.4) | -7.7 (-14.0, -1.4) | **0.018** |
| P-value (within group) | **0.001** | 1.000 |  |  |
|  |  |  |  |  |
| Stroop colour testing |  |  |  |  |
| - Baseline | 76.2 (14.5) | 76.8 (11.4) | -0.6 (-6.7, 5.5) | 0.840 |
| - 3 months | 78.7 (15.4) | 77.1 (14.8) | 1.6 (-5.5, 8.7) | 0.655 |
| - Difference | 2.6 (8.2) | 0.4 (9.4) | 2.2 (-1.9, 6.4) | 0.292 |
| P-value (within group) | 0.079 | 0.812 |  |  |
|  |  |  |  |  |
| Stroop colour testing (with words) |  |  |  |  |
| - Baseline | 37.8 (14.4) | 42.9 (12.6) | -5.1 (-11.4, 1.3) | 0.116 |
| - 3 months | 43.7 (16.6) | 44.6 (11.8) | -0.9 (-7.9, 6.0) | 0.784 |
| - Difference | 5.9 (9.6) | 1.7 (7.6) | 4.1 (0.1, 8.2) | **0.046** |
| P-value (within group) | **0.001** | 0.162 |  |  |
